# The Beginning of the End: A Chromosomal Assembly of the New World Malaria Mosquito Ends with a Novel Telomere

**DOI:** 10.1534/g3.120.401654

**Published:** 2020-09-03

**Authors:** Austin Compton, Jiangtao Liang, Chujia Chen, Varvara Lukyanchikova, Yumin Qi, Mark Potters, Robert Settlage, Dustin Miller, Stéphane Deschamps, Chunhong Mao, Victor Llaca, Igor V. Sharakhov, Zhijian Tu

**Affiliations:** * Department of Biochemistry, Virginia Tech, Blacksburg, VA24061; † Fralin Life Science Institute, Virginia Tech, Blacksburg, VA24061; ‡ Department of Entomology, Virginia Tech, Blacksburg, VA24061; § Genetics Bioinformatics and Computational Biology PhD program, Virginia Tech, Blacksburg, VA 24061; ** Institute of Cytology and Genetics SB RAS, Novosibirsk, Russia; †† Advanced Research Computing, Virginia Tech, Blacksburg, VA24061; ‡‡ MR4 Program, Center for Disease Control Foundation CDCF, Atlanta, GA30329; §§ Corteva Agriscience, Johnston, IA 50131; *** Biocomplexity Institute & Initiative, University of Virginia, Charlottesville, VA 22911; ††† Department of Genetics and Cell Biology, Tomsk State University, Tomsk, Russia

**Keywords:** Genome assembly, *Anopheles albimanus*, Oxford Nanopore, Hi-C, Bionano

## Abstract

Chromosome level assemblies are accumulating in various taxonomic groups including mosquitoes. However, even in the few reference-quality mosquito assemblies, a significant portion of the heterochromatic regions including telomeres remain unresolved. Here we produce a *de novo* assembly of the New World malaria mosquito, *Anopheles albimanus* by integrating Oxford Nanopore sequencing, Illumina, Hi-C and optical mapping. This 172.6 Mbps female assembly, which we call AalbS3, is obtained by scaffolding polished large contigs (contig N50 = 13.7 Mbps) into three chromosomes. All chromosome arms end with telomeric repeats, which is the first in mosquito assemblies and represents a significant step toward the completion of a genome assembly. These telomeres consist of tandem repeats of a novel 30-32 bp Telomeric Repeat Unit (TRU) and are confirmed by analyzing the termini of long reads and through both chromosomal *in situ* hybridization and a *Bal*31 sensitivity assay. The AalbS3 assembly included previously uncharacterized centromeric and rDNA clusters and more than doubled the content of transposable elements and other repetitive sequences. This telomere-to-telomere assembly, although still containing gaps, represents a significant step toward resolving biologically important but previously hidden genomic components. The comparison of different scaffolding methods will also inform future efforts to obtain reference-quality genomes for other mosquito species.

Mosquitoes belonging to the genus *Anopheles* transmit malaria parasites which cause one of the most devastating diseases known to mankind. In 2015, an international consortium published genome assemblies for 16 *Anopheles* species, which represent important resources and provided significant evolutionary insights (Neafsey *et al.*, 2015). The majority of these assemblies, limited by short read technologies, remain fragmented especially in repeat-rich heterochromatic regions. Recent integration of new scaffolding methods with Pacific Biosciences (PacBio) Single Molecule Real Time (SMRT) sequencing, which produces long reads, has enabled marked improvements to a few mosquito assemblies (Matthews *et al.*, 2018, Kingan *et al.*, 2019, Ghurye *et al.*, 2019). However, a substantial portion of the heterochromatic regions remain unresolved even in the best mosquito assemblies. In this study, we select the New World malaria mosquito, *Anopheles albimanus*, to explore approaches toward more inclusive mosquito genome assemblies that enable discoveries of biologically important but previously hidden genomic components. *An. albimanus* belongs to the subgenus Nyssorhynchus and is a vector of the malaria parasite *Plasmodium vivax*. This mosquito inhabits the neotropical regions of Latin America, stretching from southern United States to Peru and the Caribbean Islands (Charles and Senevet, 1953, Roberts *et al.*, 2002, Grieco *et al.*, 2006, Fuller *et al.*, 2012, Ahumada *et al.*, 2016, Cauich-Kumul *et al.*, 2018). It has one of the smallest genomes within the genus making it a suitable species for achieving our objectives. *An. albimanus* has two pairs of autosomes and one pair of sex chromosomes. Males have heteromorphic X and Y sex chromosomes. Three pairs of synaptic chromosomes in females are seen as five chromosome arms in polytene nuclei. In 2017, extensive physical mapping of this genome corrected several mis-assemblies and placed contigs onto five chromosome arms, generating a significantly improved AalbS2 assembly (Artemov *et al.*, 2017). Here we aim to produce an improved *de novo* assembly by integrating Oxford Nanopore sequencing, Illumina, Hi-C and a recent advancement in BioNano optical mapping chemistry called Direct Labeling and Staining (DLS), which results in longer molecules and produces more contiguous maps (Deschamps *et al.*, 2018, Formenti *et al.*, 2019). The resulting assembly, AalbS3, represents a significant improvement as it includes previously uncharacterized centromeric and rDNA clusters and added 7.4 Mbps sequences, most of which are repeats. More importantly, AalbS3 is organized into three chromosomes, with all five chromosome arms ending with telomeric repeats, which is the first in mosquito assemblies and indicates high quality. The discovery of a 30-32 bp Telomeric Repeat Unit (TRU), different from any known TRUs, provides an opportunity to investigate telomere function and evolution in mosquitoes.

## Materials and Methods

### Mosquito strain

The STELCA strain of *An. albimanus* was originally colonized from a population in El Salvador and deposited at the Malaria Research and Reference Reagent Resource (MR4) at Biodefense and Emerging Infections Research Resources Repository (BEI) under catalog number MRA-126. A colony of this strain was established and maintained in the insectary of the Fralin Life Science Institute at Virginia Tech. All stages were reared in a growth chamber at 27° with a 12-hour light cycle.

### Mosquito mating scheme and sample collection

**Figure 1:**
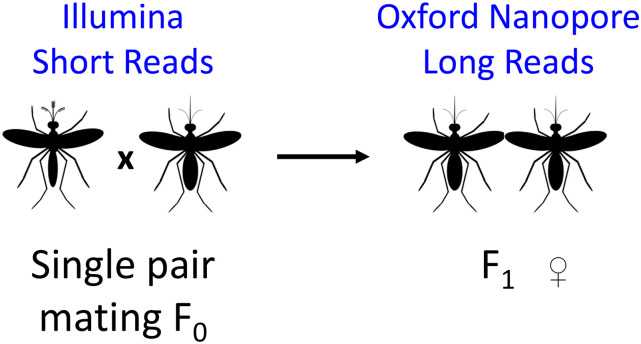
Sample and data collection scheme. The figure shows the cross of a single male and female mosquito to produce female F1 offspring. The F0 father and mother were sequenced individually using Illumina HiSeq to obtain short reads. Genomic DNA from a pool of the female F1 sibling was sequenced using three Oxford Nanopore MinION flow cells to produce long reads. Genomic DNA from a pool of the paternal half-sibling F1 males was used to generate Bionano DLS optical mapping. Oxford Nanopore reads were used to generate the contigs using Canu (Koren *et al.*, 2017) and the Canu contigs were polished using the parental Illumina reads. The polished Canu contigs were scaffolded using the Bionano DLS optical map to generate the AalbS3 assembly.

A single male mosquito and five virgin female mosquitoes were allowed to mate for 4 days in a 16 oz soup cup then given a blood meal using defibrinated sheep blood using an artificial blood-feeder. After 72 hr, eggs were collected from each female separately and reared under normal conditions. All F1 pupae from each family were sorted by sex, collected, and immediately frozen in liquid nitrogen and stored in -80°. The family of F0 female #2 was chosen for Oxford Nanopore sequencing and the F0 father and the F0 mother were collected separately, flash frozen in liquid nitrogen, and stored at -80°. The F1 male progeny of the F0 female #3 was used for Bionano DLS optical mapping. This sample collection scheme is depicted in Figure 1.

### DNA isolation

Genomic DNA was isolated from 20 female F1 pupae following a modified Qiagen Genomic Tip DNA Isolation kit (Qiagen Cat No. 10243 and 19060) protocol. For this, pupae were immediately transferred from -80° into a 50mL conical tube containing pre-prepared lysis solution consisting of 9.5mL Buffer G2 and 19μL RNase A (Qiagen Cat No. 19101). The pupae were then homogenized using a Dremel motorized homogenizer for approximately 30 sec on the lowest speed. Next, >300mAU (500μL of >600 mAU/ml, solution) Proteinase K (Qiagen Cat No. 19131) was added to the sample and incubated at 55° for 3 hr. The homogenate was then transferred into a 15mL conical tube and centrifuged at 5000x G for 15 min at 4° to remove debris. Following this, DNA was extracted following the standard Qiagen Genomic Tip protocols. The purity, approximate size, and concentration of the DNA were tested using a nanodrop spectrophotometer, 0.5% agarose gel electrophoresis, and Qubit dsDNA assay, respectively.

### Oxford Nanopore sequencing and base calling

Approximately 1μg of DNA was used to generate a sequencing library according to the protocol provided for the SQK-LSK109 library preparation kit from Oxford Nanopore. After the DNA repair and end prep and adapter ligation steps, SPRIselect bead suspension (Beckman Coulter Cat No. B23318) was used to remove short fragments and free adapters. Qubit dsDNA assay was used to quantify DNA and approximately 300-400 ng of DNA library was loaded onto a MinION flow cell. Base calling was performed using albacore with the default filtering setting of Qscore >7 (de Lannoy *et al.*, 2017) (BioProject Number PRJNA622927, BioSample SAMN14582895).

### Illumina sequencing library preparation and sequencing

Genomic DNA was isolated from F0 mother and F0 father using the QiaAMP DNA micro kit (Qiagen Cat No. 56304). Approximately 300 ng of genomic DNA was used to prepare DNA sequencing libraries for each parent following the protocol of NEBNext Ultra II FS DNA Library Prep Kit for Illumina (NEB Cat No. #E7805S/L). The libraries were sent to Novogene (https://en.novogene.com/) for sequencing. More than 50 Gb of 2x150 bp reads were obtained from the father and mother, respectively (BioProject Number PRJNA622927, BioSamples SAMN14582897 and SAMN14582898, respectively).

### Contig assembly and polishing

Quality-filtered sequences longer than 2 kbps were assembled with Canu (Koren *et al.*, 2017) using the cascades high-performance computer at the Advanced Research Computing facility of Virginia Tech. This generated a 197.39 Mbps genome consisting of 660 contigs with an N50 of 13.7 Mbps. These contigs were polished with Pilon (Walker *et al.*, 2014) for four rounds using parental Illumina short reads.

### Ultra-high molecular weight (uHMW) nuclear DNA isolation and Bionano Mapping

Ultra-high molecular weight (uHMW) nuclear DNA was isolated from approximately 40 pooled male siblings (Figure 1) using a modification of the Bionano Prep Animal Soft Tissue DNA Isolation protocol (bionanogenomics.com; document 30077). Flash-frozen pupae were homogenized with a chilled Dounce grinder in the presence of ice-cold Bionano Prep homogenization buffer. The sample was then filtered through a single 100um cell strainer, mixed with an equal part of cold 200-proof ethanol, and incubated for one hour at room temperature. Nuclei and debris were pelleted by centrifugation at 1,500xg for 5 min at 4°, followed by four wash-centrifugation cycles with homogenization buffer. The resulting pellet was embedded in three 90-ul low-melting-point agarose plugs and treated with both proteinase K and RNaseA as per manufacturer’s recommendations. Free ultra-high molecular weight nuclear DNA was recovered by melting the plugs, digesting with agarase and dialyzing against TE buffer. Data collection for optical mapping was performed in a Bionano Saphyr platform running a sample prepared according to the Direct Label and Stain (DLS) process (Bionano Genomics Cat.80005) following manufacturer’s protocols with some modifications. Approximately 500ng uHMW nDNA was incubated for 2:20 h at 37°, followed by 20 min at 70° in the presence of DLE-1 Enzyme, DL-Green and DLE-1 Buffer. The labeling reaction was killed by adding proteinase K and incubate at 50° for 1hr, followed by clean-up of the unincorporated DL-Green label. Labeled, cleaned-up DNA was then combined with a Flow/DTT buffer as per Bionano Genomics specifications and incubated overnight at 4°. After quantification, DNA was stained by adding Bionano DNA Stain to a final concentration of 1 microliter per 0.1 microgram of final DNA, loaded into a Saphyr chip flowcell. Molecules were stretched, separated, imaged and digitized using software installed in a Bionano Genomics Saphyr System and server according to the manufacturer’s recommendations (https://bionanogenomics.com/support-page/saphyr-system/). Molecules were automatically filtered with a minimum length of 150 kb and a minimum of 9 labels, resulting in a 342-Gbp subset of 1,014,877 molecules with a N50 of 373 kbp and average label density of 9.85 per 100 kbp. The molecules were assembled into maps by the Bionano Solve Version 3.3, RefAligner Version 7989 and Pipeline Version 7981 software. The used parameters were “non-haplotype without extend and split”, no CMPR cuts, and 200 Mbp expected genome size. The resulting assembly included 29 maps with an N50 of 51 Mbp and a total combined length of 223 Mbp. Bionano (BNG) maps were aligned to the described sequence assembly to generate hybrid scaffolds using the Bionano Solve V3.3 suite. The alignment produced only 8 scaffolds, with a total scaffold length of 184.23 Mbp, including a 11.8 Mbp map with little corresponding sequence from the female sequence assembly. Sequence was manually curated to trim sequence overlaps, remove secondary contigs due to heterozygosity, break up mis-assemblies and ultimately validate final sequence.

### Hi-C scaffolding

**Figure 2:**
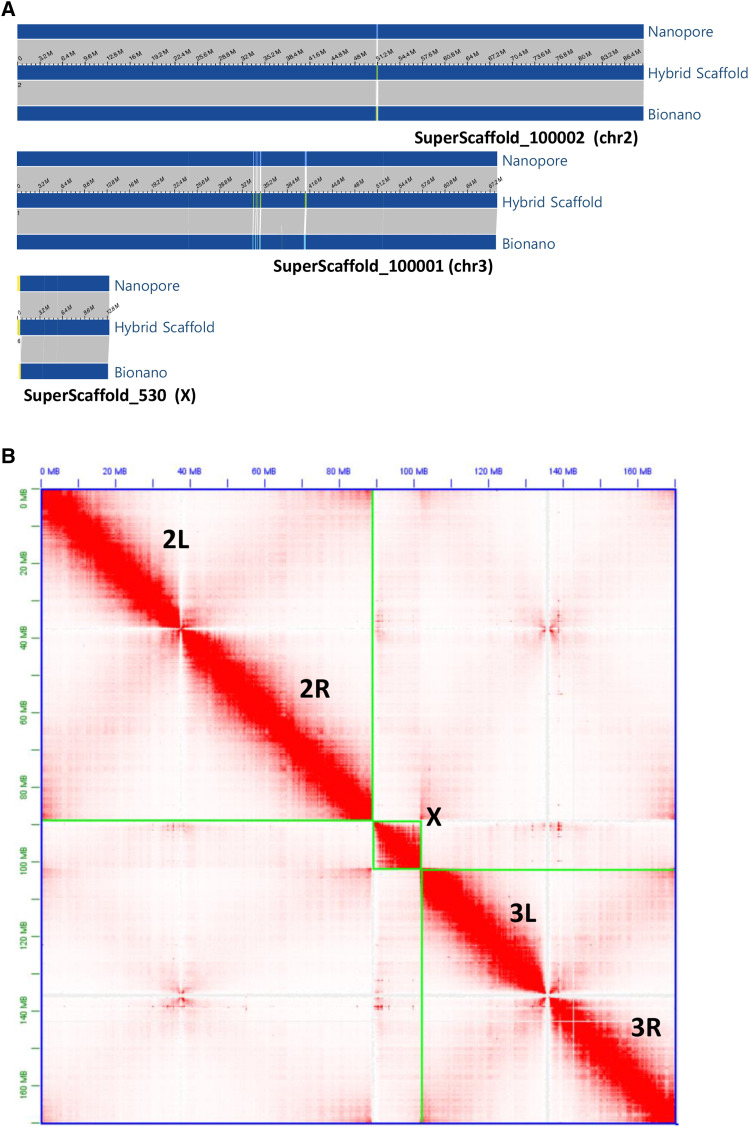
A) Chromosome level hybrid scaffolds produced by aligning polished Nanopore contigs to Bionano optical maps. The orientation of the hybrid scaffolds as shown in the figure from left to right: Chr2 R to L, Chr3 L to R, and X centromere to telomere. B) Hi-C contact matrix for the three chromosomes of the hybrid assembly. The order of the three *An. albimanus* chromosomes, shown in green boxes, are 2, X, and 3. Note that the final AalbS3 assembly includes the rDNA clusters and centromeric repeats which are not shown in the figure.

Hi-C Illumina reads were available from a multi-species Hi-C analysis project (PRJNA615337) that included 2 replicates of *An. albimanus* mixed embryo samples (SAMN14451359). Hi-C reads were aligned to either the polished Canu contigs for independent scaffolding or to the Bionano scaffolds (Figure 2A) for validation (Figure 2B). 3D-DNA pipeline was employed to assemble the *An. albimanus* genome *de novo* using the generated Hi-C data set. Misassemblies were identified and fixed manually using assembling mode in Juicebox software (Durand *et al.*, 2016). The *An. albimanus* physical genome map (Artemov *et al.*, 2017) was used to assess the assemblies

### Quality and heterozygosity assessment

The completeness of the AalbS2 and AalbS3 assemblies were measured using a Benchmarking Universal Single Copy Orthologs (BUSCO) test (Seppey *et al.*, 2019, Waterhouse *et al.* 2019). Nucleotide accuracy of the assembly was assessed by calculating Quality Value (QV) scores as described in Berlin *et al.* (2015) and Solares *et al.* (2018). To estimate heterozygosity, Illumina reads from each of the parents were mapped to the AalbS3 assembly using BWA (Li and Durbin 2009) with default parameters. GTAK (Poplin *et al.*, 2018) was then used, with default parameters, to identify SNP and indel sites. Sites that showed a Genotype Quality greater than 50 were counted as sites with heterozygosity. Heterozygosity rates were calculated for each chromosome as the total number of heterozygous sites in a chromosome divided by the length of the chromosome excluding ambiguous N bases.

### Repeat analysis

The AalbS3 assembly was used to uncover repeat sequences using RepeatModeler (http://www.repeatmasker.org/RepeatModeler/) with default settings. The repeat library generated by RepeatModeler was then used to mask either the AalbS2 or AalbS3 assembly, by using RepeatMasker (http://www.repeatmasker.org) with default settings, for comparison of repeat content.

### Chromosome preparation and fluorescence in situ hybridization

Polytene chromosome and mitotic chromosome preparations were made as previously described (Sharakhov 2014). Salivary glands were dissected from one 4^th^ instar larvae, which was stored in Carnoy’s solution (Methanol: Glacial acetic acid = 3:1) and used for one polytene chromosome preparation. Isolated salivary glands were bathed in a drop of 50% propionic acid for 5 min and squashed under a 22 × 22 mm cover slip. Mitotic chromosome preparations were made from leg and wing imaginal discs of early 4^th^ instar larvae. A fresh drop of hypotonic solution was added to the preparation of imaginal discs for 10 min, followed by fixation in a drop of modified Carnoy’s solution (ethanol:glacial acetic acid, 3:1) for 1 min. Next, a drop of freshly prepared 50% propionic acid was added, and the imaginal discs were covered with a 22 × 22 mm coverslip. The quality of chromosomal preparation was assessed with an Olympus CX41 phase-contrast microscope (Olympus America Inc., Melville, NY). High-quality chromosome preparations were flash frozen in liquid nitrogen, coverslips were quickly removed by a razor blade and preparations were immediately placed in cold 50% ethanol. After that, preparations were dehydrated in an ethanol series (50%, 70%, 90%, and 100%), air-dried and stored at room temperature (RT) until the use for FISH. To prepare the probe, an oligonucleotide was designed based on sequences of the telomeric satellite albi_telomere1: GTTCCTATAGCTTCTCTCACTCAAGTAGCCT and labeled with 3′- end- Cyanine3 fluorochrome (Sigma-Aldrich, St. Louis, MO, USA) The sequence of the oligonucleotide probe is AGGCTACTTGAGTGAGAGAAGCTATAGGAAC [Cyanine3]. FISH was performed as previously described (Sharakhov, 2014, Timoshevskiy *et al.* 2012) with modifications. Briefly, slides with good chromosome preparations were washed in 1×phosphate-buffered saline (PBS) for 20 min and fixed in 3.7% formaldehyde for 1 min at RT. Slides were then washed in 1×PBS briefly and dehydrated in a series of 70%, 90%, and 100% ethanol for 5 min at RT. Then, 10 µl of 100 μM oligonucleotide probes diluted in the hybridization buffer, including 1200 μl deionized formamide, 0.2 g Dextran Sulfate, 120 μl 20×SSC and 500 μl H 2 O, were added to the preparations. After heating at 73° for 5 min, slides were incubated at 37° overnight. After washing in 1×SSC at 60° for 5 min and 4×SSC/NP40 solution at 37° for 10 min, slides were briefly washed in 1×PBS and incubated in YOYO-1 for 10 min at RT. After rinsing in 1×PBS, preparations were counterstained with an antifade solution (Life Technologies, Carlsbad, CA, USA) and kept in the dark for at least 2 hr before visualization using a ZEISS Axio Imager 2 fluorescent microscope (Zeiss, Oberkochen, Germany) with a connected Axiocam 506 mono digital camera (Zeiss, Oberkochen, Germany).

### Bal31 Sensitivity Assay

HMW genomic DNA was extracted from 50 *Anopheles albimanus* following an SDS-based method as previously described (Xia *et al.*, 2019). The *Bal*31 Sensitivity Assay was performed based on the protocol of (Richards and Ausubel 1988, Yang *et al.*, 2017) with several modifications. Briefly, 2 µg of HMW genomic DNA was treated with *Bal*31 exonuclease for the prescribed amount of time (0, 30, 60, 120, and 240 min) followed by inactivation by the addition of ethylene glycol tetraacetic acid (EGTA) to a final concentration of 20 mM and incubated at 65° for 5 min. The digested DNA was recovered using a phenol-chloroform extraction and ethanol precipitation as described above. The recovered DNA was treated with *Xba*I for two hours at 37° and inactivated by heating at 65° for 20 min. Following this, 20 µL of each sample was analyzed by Pulsed-Field Gel Electrophoresis (PFGE) for 8 hr at 14° (6 V/cm, 10-50 sec switch time). PFGE-separated DNA was transferred onto Hybond-N+ charged nylon membrane by downward capillary transfer. Hybridization of the digoxegenin (DIG)-labeled 31bp oligonucleotide probe and detection was carried out following the DIG High-Prime Labeling and Detection Starter Kit (Roche SKU 11745832910). 100 µl of extraction buffer (200 mM Tris-HCl, pH 8.0; 0.5% Sodium dodecyl sulfate; 250 mM NaCl; 50mM EDTA)

### Data availability

The AalbS3 *Anopheles albimanus* genome assemblies: NCBI Genome database https://www.ncbi.nlm.nih.gov/genome under Umbrella BioProject PRJNA655695. The primary assembly is GCA_013758885.1 under BioProject PRJNA622927 and the alternative is GCA_014083485.1 under BioProject PRJNA637113. Raw data deposited in NCBI Sequencing Read Archive (SRA) https://www.ncbi.nlm.nih.gov/sra/ under PRJNA622927: F1 females ONT SAMN14582895, F0 father SAMN14582897, F0 mother SAMN14582898. Bionano molecules are deposited in NCBI Sequencing Read Archive (SRA) https://www.ncbi.nlm.nih.gov/sra/ under PRJNA622927: F1 males SAMN14582896. Hi-C sequencing reads are deposited in NCBI Sequencing Read Archive (SRA) https://www.ncbi.nlm.nih.gov/sra/ under PRJNA615337: 2 replicates *Anopheles albimanus* SAMN14451359. Supplemental material available at figshare: https://doi.org/10.25387/g3.12130794.

## Results

### Assembly of the Anopheles albimanus genome

We performed ONT sequencing using gDNA isolated from sibling females from a single-pair mating (Figure 1). We also sequenced both parents using Illumina for polishing. Raw ONT reads were base called using albacore and quality-filtered sequences were assembled with Canu (Koren *et al.*, 2017) applying a read length filter, excluding those shorter than 2 kbps. This generated a 197.39 Mbps genome consisting of 660 contigs with an N50 of 13.7 Mbps. These contigs were polished with Pilon (Walker *et al.*, 2014) using parental Illumina short reads. To scaffold these contigs, we performed optical mapping using ultra high molecular weight genomic DNA isolated from paternal half-sibling males (Figure 1). Direct Label and Stain (DLS) chemistry was used for this purpose, which produced 342 Gbps of molecules with a N50 of 373 kbps. The resulting Bionano assembly included 29 maps with an N50 of 51 Mbp and a total length of 223 Mbp. Bionano maps were then aligned to the previously mentioned 197.39 Mbps sequence assembly to generate hybrid scaffolds. The alignment produced only 8 scaffolds, with a total scaffold length of 184.23 Mbp. An 11.8 Mbp map had no or little correspondence with sequences from the female sequence assembly. This 11.8 Mbp map may correspond to the Y chromosome as males were used for Bionano mapping. A 5.2 Mb scaffold corresponds to the circular genome of *Serratia marcescens*, a known bacterium in the mosquito microbiome (Gonzalez-Ceron *et al.*, 2003). The remaining sequences were manually curated to trim sequence overlaps, remove secondary contigs due to heterozygosity, break up mis-assemblies. The curated assembly consists of five superscaffolds including the three chromosomes X, 2, and 3 (Figure 2A), a single X-linked rDNA cluster of approximately 803 kb and an unplaced sequence of ∼238 kb. The Hi-C contact matrix of the three chromosomes (Figure 2B) indicates good quality. Two scaffolds representing centromeric regions of chromosome 2 and 3 were added to the above five Bionano superscaffolds, resulting in the final 172.6 Mbps AalbS3 assembly (Table 1, Figure 2). The two centromeric sequences contain highly repetitive tandem repeats (Table S1, File S1) and they were produced by Hi-C scaffolding of the polished contigs produced by Canu, as described below. The AalbS3 assembly more than doubled the content of transposable elements and other repetitive sequences (Table S2, File S2). The QV score (Berlin *et al.* 2015, Solares *et al.* 2018) of the AalbS3 assembly is 35.839, corresponding to 99.95% nucleotide accuracy. The completeness of the assembly was measured using a Benchmarking Universal Single Copy Orthologs (BUSCO) test (Seppey *et al.*, 2019, Waterhouse *et al.*, 2019) and scored a 99.0% with 98.2% of genes represented in this genome as complete and single copy and 0.8% as complete and duplicated (Table 1 and Table S3). By mapping Illumina reads from each of the parents to the AalbS3 assembly, the levels of heterozygosity were also calculated for each chromosome, which range from 0.0004 to 0.0011 (Table S4, Figure S1).

**Table 1:** Comparison overview of AalbS2 and AalbS3

	AalbS2	AalbS3
Total Length	173.3 Mb	172.6 Mb
Total Length Excluding Ns	163.5 Mb	170.9 Mb
Contig N50	0.2 Mb	13.7 Mb
Number of scaffolds	5 chromosome arms plus 196 scaffolds	3 chromosomes plus 4 other scaffolds
Scaffold N50	37.9 Mb	89 Mb
Telomere	Not detected	Found at the end of all five chromosome arms
rDNA cluster	Not detected	An 800 kb scaffold containing the rDNA cluster
BUSCO	98.9%	99.0%
Total Repeat Content	2.20%	5.06%

### Comparison of Hi-C and Bionano scaffolding

We also produced an assembly by scaffolding the polished Canu contigs using Hi-C data, enabling informative comparisons between the two leading scaffolding technologies, Bionano and Hi-C. Using Bionano maps, the Hi-C assembly was first examined for misassemblies and several chimeric mis-joins and insertions were observed that were not present in the Bionano assembly. We also assessed the quality of each assembly using physical mapping data previously used to produce the AalbS2 scaffolds (Artemov *et al.*, 2017). Gene sequences used as probes for FISH were aligned to the Bionano and Hi-C assemblies, respectively, and their order and orientation were compared (Table S5). We found that chromosomes X and 2 were in perfect concordance with respect to order and placement in both Hi-C and Bionano assemblies. Both Bionano and Hi-C assemblies disagreed with the FISH results in two segments on chromosome 3, indicating either chromosomal variations between samples or mistakes in FISH. Four FISH probes were not placed on the Hi-C assembly but were placed correctly in the Bionano assembly. Thus, we conclude that the overall quality of the Bionano assembly was better than the Hi-C assembly, perhaps due to the ability of the new DLS chemistry to produce ultra-long optical mapping molecules (Jiao *et al.*, 2017). However, Bionano had difficulties assembling the tandem repeats of the centromeric region of chromosome 2 into the chromosomal scaffolds. This is likely due to an overall lack of labeling sites on the molecules. Thus, Bionano is a preferred primary scaffolding technology in this study but it should be supplemented with Hi-C for long-range scaffolding of repetitive regions that have either low or overly dense signals, similar to the strategy used in recent assemblies of plant genomes (Jiao *et al.*, 2017).

### Discovery of a novel telomeric sequence and validation by analyzing the termini of long reads

**Figure 3:**
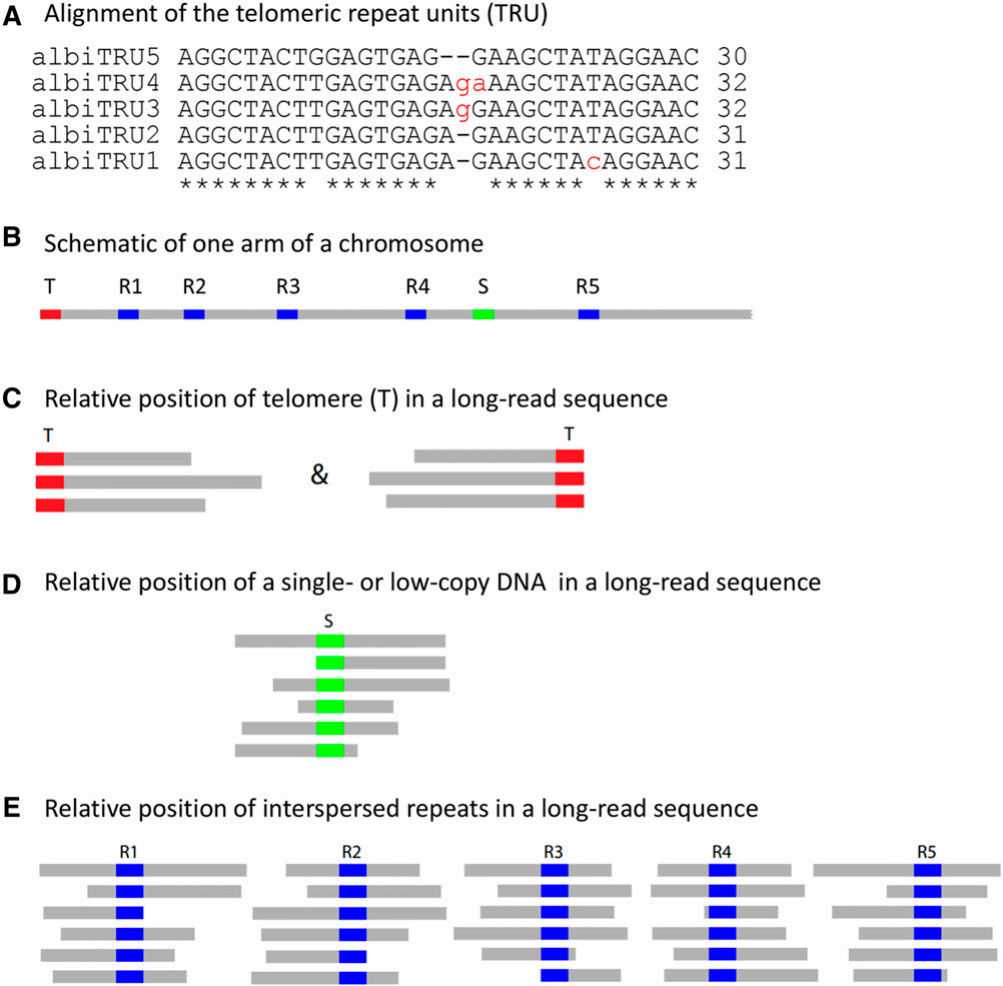
Alignments of variants of the telomeric repeat unit (TRU, panel A) and the principle underlying the strategy to verify telomere sequences using long reads (panels B-E). T: telomere, which is a tandem repeat of TRUs, or (TRU)n; S: single- or low-copy DNA sequences; R1-R5: five copies of an interspersed repeat.

We observed tandemly repeating units of 30-32bp (Figure 3A) at the ends of the chromosomes 2L, 2R, 3L, 3R and X in our AalbS3 assembly, leading us to hypothesize that these are telomeric repeat units (TRUs). As shown in Figure 3A, the 30-32 bp TRUs are very similar to each other and they form a tandemly repeated region of kilobases in length. These putative TRUs were not observed in the AalbS2 assembly as the ends of its five chromosome arms are 27-69 kb inside the chromosome arms of the AalbS3 assembly. The 30-32bp telomeric repeat units described in this study are different from all reported telomeric repeats (http://telomerase.asu.edu/sequences_telomere.html), including the only *Anopheles* telomeric sequence found in *Anopheles gambiae* (Biessmann *et al.*, 1996). We selected 97,921 error-corrected Oxford Nanopore reads longer than 40 kb, equivalent to ∼32x genome coverage, to validate that the AalbS3 assembly is indeed able to extend to telomeres. As shown in Figure 3B–E, reads that contain a true telomere should either start or end with the telomeric sequences while reads that contain other sequences inside the chromosomes should not all start or end with the same sequence or repeat unit. We searched the 97,921 long reads for sequences that contain any of the five TRUs (BLASTN, evalue 1e-5) and identified 149 reads. Not all 149 are expected to be telomeric sequences as the TRUs also show similarity to two regions that are 77.8kb and 78.1 kb away from the 2L and X telomeres, respectively. Up to 100% identity is observed between repeat units in the 2L subtelomeric region and TRU1 while up to 97% identity is observed between the repeat units in the X subtelomeric region and TRU1. We filtered out 65 sequences that are derived from these regions. The remaining 84 long reads (File S3) were analyzed using a combination of RepeatMasker (default parameters, library being the monomers or dimers of TRUs), BLASTN (evalue 1e-5), and manual inspection. As shown in Table 2, all but one of the 84 reads either start or end with the TRU, and the only exception was likely a chimera, with ∼39kb matching 2R and ∼23 kb matching X. Thus, analysis of the long reads strongly support that the AalbS3 assembly indeed extended to the telomeres in all five chromosome arms.

**Table 2:** ONT Telomeric long reads

Chromosome Arm	Number of Reads beginning with TRU	Number of Reads terminating with TRU	Average Length of Reads with TRU
X	6	4	60.56
2R	6	9	59.86
2L	4	19	53.72
3R	17	7	54.53
3L	4	7	55.75

Only reads longer than 40 kb were analyzed.

### Experimental verification of the telomeric sequences

**Figure 4:**
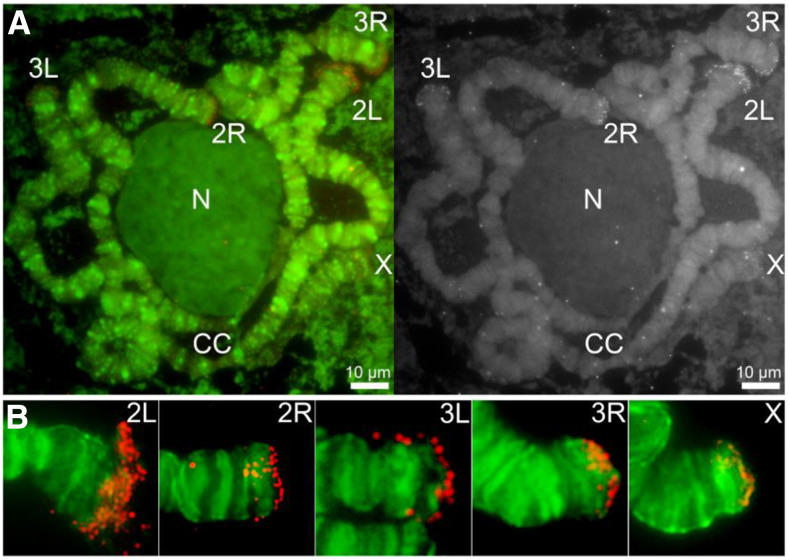
Fluorescence *in situ* hybridization and mapping of the *An. albimanus* telomeric oligonucleotide probe on polytene chromosomes from the 4^th^ instar larva. (A) Left panel: Chromosomes hybridized with Cy3-labeled oligonucleotide probe (red) and counterstained with the fluorophore YOYO-1 (green). Right panel: An inverted grayscale image of the FISH results of the oligonucleotide probe. Chromosome arms are labeled as X, 2R, 2L, 3R, and 3L; the chromocenter is labeled by CC; the nucleolus is labeled by N. Scale bar – 10μm.(B). An enlarged view of representative chromosome arms hybridized with Cy3-labeled oligonucleotide probe (red).

**Figure 5:**
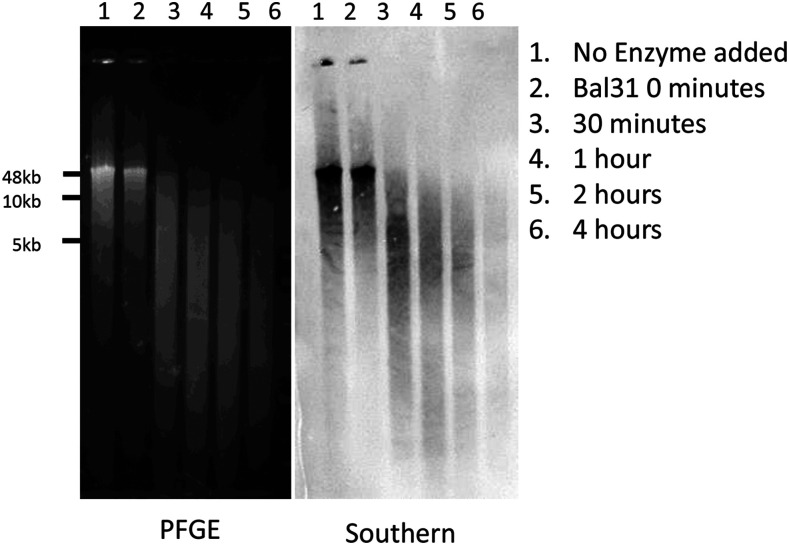
*Bal31* PFGE and Southern Blot. HMW genomic DNA from *An. albimanus* pupae was digested with *Bal*31 exonuclease at time point increments followed by restriction enzyme digestion with *Xba*I then separated by Pulsed-Field Gel Electrophoresis (left). The separated DNA was transferred to charged Nylon membrane via downward capillary action, hybridized with a DIG- labeled telomeric probe, and detected using chemiluminescence (right).

To further validate the novel 30-32 bp TRUs, we performed fluorescence *in situ* hybridization (FISH) on polytene (Figure 4) and mitotic (Figure S2) chromosomes to validate their location. A complementary oligonucleotide probe was designed based on one of the TRUs, albi_telomere1 (GTTCCTATAGCTTCTCTCACTCAAGTAGCCT). The telomeric oligonucleotide probe hybridized to all tips of chromosome arms, including 2L, 2R, 3L, 3R and X. Although the X chromosome has two telomeres, our FISH with polytene chromosomes detected hybridization signals only at one end of the X chromosome. The other end of the X chromosome telomere is associated with heterochromatin and embedded in the chromocenter. Each arm was recognized based on the published *An. albimanus* cytogenetic map (Artemov *et al.*, 2017). The FISH signals from telomeric repeats are present at the tips of all telomeric ends. The intensity of the signals was higher on autosomes than on the X chromosome suggesting a possibly smaller number of copies on the sex chromosome. In addition, we used a modified *Bal*31 exonuclease sensitivity assay (Richards and Ausubel 1988, Yang *et al.*, 2017) to further validate these telomeric sequences. For this, HMW genomic DNA was digested with *Bal*31 to shorten ends of DNA, which was subsequently fragmented with a restriction enzyme, and detected via oligonucleotide probe hybridization by Southern Blotting (Figure 5). The telomeric probe hybridizes to *Bal*31-sensitive sites as indicated by progressive shortening of the detected sequences, consistent with the TRUs having a terminal position on the chromosomes.

## Discussion

In this work we have produced the AalbS3 assembly for *Anopheles albimanus*, an important vector of malaria in Central and South America. By extending to telomeres in all chromosomal arms, assembling rDNA and centromeric clusters, and recovering other repetitive sequences, the AalbS3 assembly represents a significant improvement to the previously reported AalbS2, which was one of the best *Anopheles* assemblies (Neafsey *et al.*, 2015, Artemov *et al.*, 2017). Our experience provides insights that may inform future efforts to generate reference-quality *Anopheles* genome assemblies. First, we have shown that Oxford Nanopore sequencing is an attractive platform to generate long reads for assembling mosquito genomes. Prior to this report, only PacBio has been used as the long-read technology in place of Illumina to generate high quality mosquito assemblies (Matthews *et al.*, 2018, Ghurye *et al.*, 2019, Kingan *et al.*, 2019). The affordability and portability of some ONT platforms are attractive features when considering future vector sequencing projects especially for resource-limited areas or field stations. For example, we now routinely obtain >15 Gbases (60-80X coverage of an *Anopheles* genome) of long reads per ONT MinION run at a cost of approximately $600 including flow cell and library preparation. Ten Gbases of Illumina reads for polishing now cost less than $200. Second, our experience also highlights the relative effectiveness and accuracy of Hi-C and Bionano for scaffolding contigs to generate chromosome-scale assemblies. We have shown that the Bionano DLS optical mapping is a more accurate primary scaffolding method than the Hi-C method commonly used in mosquito assemblies (Dudchenko *et al.*, 2017, Matthews *et al.*, 2018, Ghurye *et al.*, 2019). However, Bionano should be supplemented with Hi-C for long-range scaffolding of repetitive regions that have either low or overly dense DLS signals (Jiao *et al.*, 2017). It is important to note that Hi-C is not ideal for organizing centromeric repeats. However, the two Hi-C-based centromeric scaffolds provides a starting point for future research on *An. albimanus* centromeres.

An improved assembly provides a better genomic resource and can profoundly impact investigations into the molecular genetics and evolution of the species. For example, information on previously uncharacterized centromeric repeats will facilitate chromosomal analysis during mitosis and meiosis. Furthermore, recovering a large number of repeats will inform analysis of genome structure and repeat-associated small RNAs. Most importantly, we discovered novel telomeric repeats present at chromosomal termini. These differ in both sequence and structure from telomeric repeats reported in other dipteran species, which may indicate a novel telomere synthesis or maintenance mechanism and provide new evolutionary insights. The approach we used to validate the telomeres took advantage of the ONT long reads as TRU-containing long reads should either begin or end with TRU sequences. This method could be generally applicable for the discovery and validation of telomeres in diverse organisms.

There are generally three known mechanisms that maintain telomere length in eukaryotes. The most common mechanism used by many organisms including humans, employs a telomerase consisting of a reverse transcriptase and an RNA template to extend short tandem repeats at chromosome ends (Morin 1989). The second, described in *Drosophila*, involves assembly of HeT-A and TART retrotransposon arrays at chromosomal termini (Traverse and Pardue, 1988, Valgeirsdóttir *et al.* 1990, Levis *et al.*, 1993). The third mechanism, as described in *An. gambiae* through a serendipitous discovery of a transgene inserted into the telomeric regions, possibly relies on unequal crossover of telomeric repeats that are hundreds of bases long (Biessmann *et al.*, 1996, Roth *et al.*, 1997). Contrasting the 820bp satellite repeat units reported in *An. gambiae*, here we describe much smaller 30-32bp TRUs at the chromosome ends of *An. albimanus*. BLAST analysis indicates that the *An. albimanus* TRUs do not resemble the *An. gambiae* telomeric satellite nor the *Drosophila* telomeric retrotransposons. A study using a *D. melanogaster* strain that produces abnormally long telomeres (Siriaco *et al.*, 2002) showed that longer telomeres do not necessarily confer a longer life span (Walter *et al.*, 2007). It is not yet clear how telomeres are maintained by the novel TRUs in *An. albimanus* and whether telomeric lengths are correlated with the life span in mosquitoes. The discovery of the *An. albimanus* TRUs will facilitate the development of reliable methods to quantify telomeric length (*e.g.*, Cawthon 2009, Vaquero-Sedas and Vega-Palas 2014), which will enable the detection of natural variations in telomere length and the investigation of the correlation between telomere length and mosquito life span. Such information is potentially important to disease transmission as only female mosquitoes that survive long enough to allow the completion of parasite development are responsible for pathogen transmission (Macdonald, 1957, Smith and Ellis McKenzie 2004).
